# Supplementary material for: A high-resolution atlas of the brain predicts lineage and birth order underlying neuronal identity
Source: Cell Genom. Author manuscript; Available in PMC 2026 Feb 19. (PMC7618732; doi:10.1016/j.xgen.2025.101103)
Supplement: Supplementary file 1 [file EMS212294-supplement-Supplemental_information.zip › 1-s2.0-S2666979X25003593-mmc1.pdf]

Cell Genomics, Volume *6*

Supplemental information

A high-resolution atlas of the brain predicts lineage and birth order underlying neuronal identity

Aaron M. Allen, Megan C. Neville, Tetsuya Nojima, Faredin Alejevski, Devika Agarwal, David Sims, and Stephen F. Goodwin

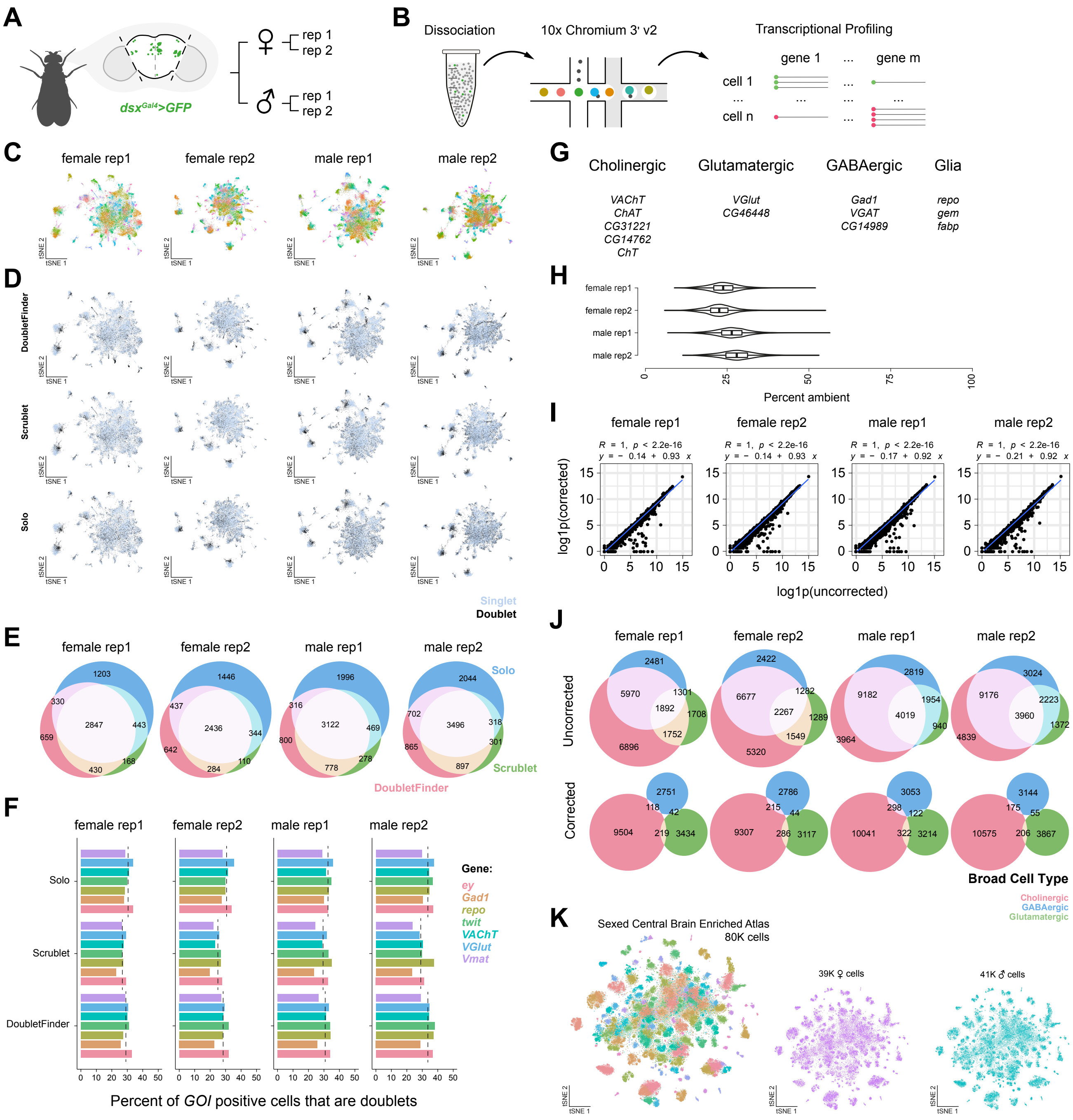
A
dsxGal4>GFP
rep 1
rep 2
rep 1
rep 2
B
Dissociation
10x Chromium 3' v2
Transcriptional Profiling
gene 1
gene m
cell 1
cell n
C
female rep1
female rep2
male rep1
male rep2
tSNE 1
tSNE 2
D
DoubletFinder
Scrublet
Solo
Singlet
Doublet
E
female rep1
female rep2
male rep1
male rep2
Solo
Scrublet
DoubletFinder
1203
330
2847
443
659
430
168
1446
437
2436
344
642
284
110
1996
316
3122
469
800
778
278
2044
702
3496
318
301
865
897
F
female rep1
female rep2
male rep1
male rep2
Solo
Scrublet
DoubletFinder
Gene:
ey
Gad1
repo
twit
VAChT
VGlut
Vmat
0 10 20 30 40 50
Percent of GOI positive cells that are doublets
G
Cholinergic
Glutamatergic
GABAergic
Glia
VAChT
ChAT
CG31221
CG14762
ChT
VGlut
CG46448
Gad1
VGAT
CG14989
repo
gem
fabp
H
female rep1
female rep2
male rep1
male rep2
0
25
50
75
100
Percent ambient
I
female rep1
female rep2
male rep1
male rep2
R = 1, p < 2.2e-16
y = − 0.14 + 0.93 x
R = 1, p < 2.2e-16
y = − 0.14 + 0.93 x
R = 1, p < 2.2e-16
y = − 0.17 + 0.92 x
R = 1, p < 2.2e-16
y = − 0.21 + 0.92 x
log1p(corrected)
log1p(uncorrected)
J
female rep1
female rep2
male rep1
male rep2
Uncorrected
Corrected
2481
5970
1301
1892
1708
1752
6896
2422
6677
1282
2267
1289
1549
5320
2819
9182
1954
4019
940
3964
3024
9176
2223
3960
1372
4839
2751
118
42
9504
219
3434
2786
215
44
9307
286
3117
3053
298
122
10041
322
3214
3144
175
55
10575
206
3867
Broad Cell Type
Cholinergic
GABAergic
Glutamatergic
K
Sexed Central Brain Enriched Atlas
80K cells
39K ♀ cells
41K ♂ cells

Figure S1

Figure S1. Generation of sexed central brains scRNA-seq dataset.

Related to Figure 1.

(**A**) Generation of sexed central brain scRNA-seq atlas: central brains expressing *doublesex* driven EGFP were dissected, dissociated, and analyzed.
(**B**) 10x Chromium 3' v2 workflow for sexed single-cell atlas.
(**C**) t-SNEs of individual replicates with cell types defined by unique colors.
(**D**) Singlets (light blue) vs doublets (black) across replicates comparing alternative identification methods (DoubletFinder, Scrublet, and Solo).
(**E**) Euler diagrams comparing doublets identified between methods per replicate.
(**F**) Percent of GOI positive cells that are doublets by method, per replicate.
(**G**) Sets of cell-type-specific, mutually exclusive marker genes for broad cell types used for estimating ambient contamination.
(**H**) Combination violin boxplots of the estimated percent ambient contamination in each replicate.
(**I**) Per replicate correlation of pseudo-bulked uncorrected and corrected gene counts (UMI).
(**J**) Euler diagrams comparing the co-expression of mutually exclusive markers for broad cell types with uncorrected (top) and corrected (bottom) gene expression profiles between replicates.
(**K**) t-SNEs of sexed central brains atlas with cell types defined by unique colors (left) and by sex (right).

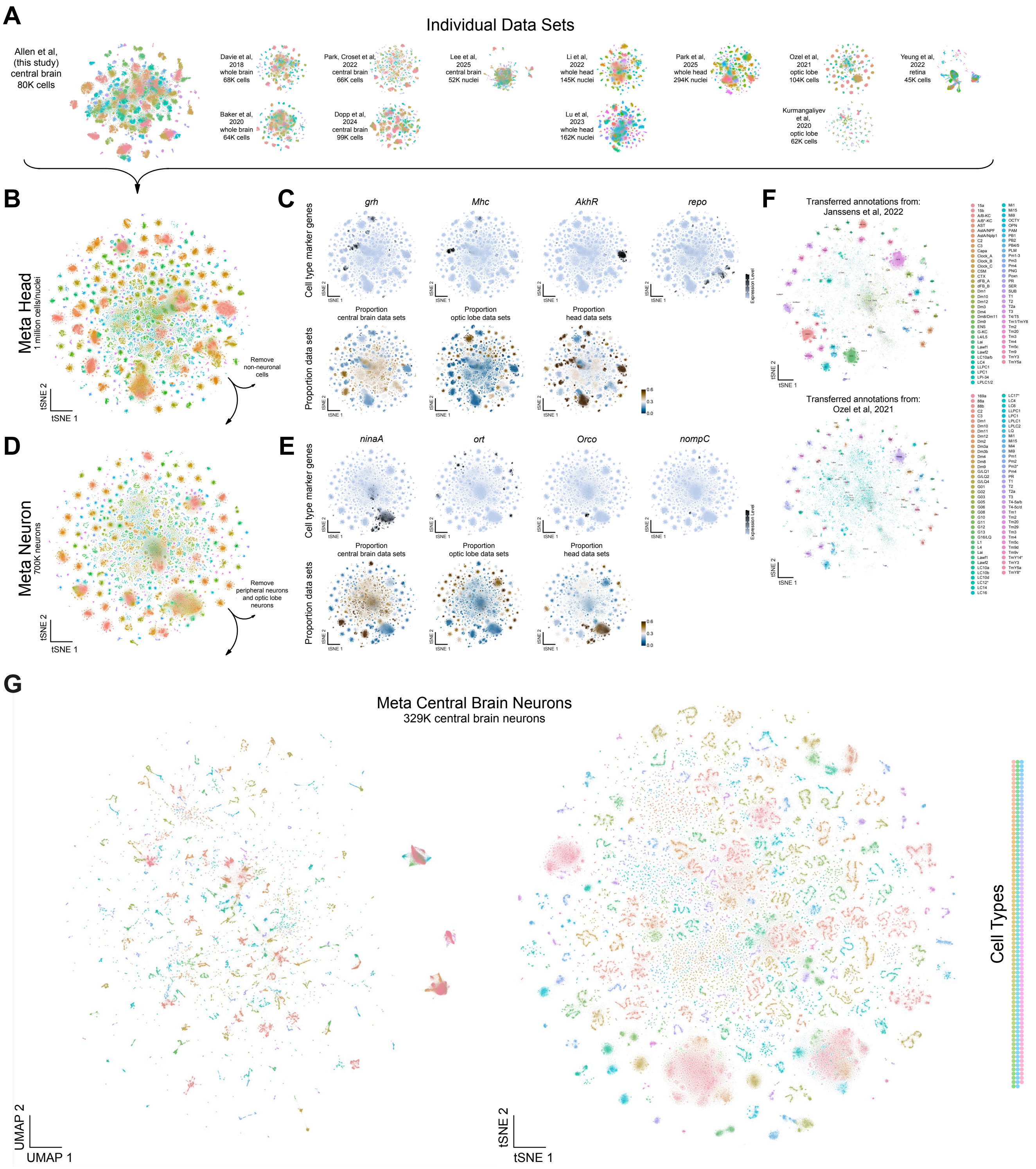
A
Individual Data Sets
Allen et al, (this study) central brain 80K cells
Davie et al, 2018 whole brain 68K cells
Park, Croset et al, 2022 central brain 66K cells
Lee et al, 2025 central brain 52K nuclei
Li et al, 2022 whole head 145K nuclei
Park et al, 2025 whole head 294K nuclei
Ozel et al, 2021 optic lobe 104K cells
Yeung et al, 2022 retina 45K cells
Baker et al, 2020 whole brain 64K cells
Dopp et al, 2024 central brain 99K cells
Lu et al, 2023 whole head 162K nuclei
Kurmangaliyev et al, 2020 optic lobe 62K cells
B
Meta Head
1 million cells/nuclei
Remove non-neuronal cells
C
Cell type marker genes
grh
Mhc
AkhR
repo
Expression Level
Proportion data sets
Proportion central brain data sets
Proportion optic lobe data sets
Proportion head data sets
D
Meta Neuron
700K neurons
Remove peripheral neurons and optic lobe neurons
E
ninaA
ort
Orco
nompC
F
Transferred annotations from: Janssens et al, 2022
Transferred annotations from: Ozel et al, 2021
tSNE 1
tSNE 2
G
Meta Central Brain Neurons
329K central brain neurons
UMAP 1
UMAP 2
Cell Types

Figure S2

Figure S2. Integration and cell type identification within the meta-head and meta-neuron atlases.

Related to Figure 1.

(**A**) t-SNEs of individual datasets[S1-11] reprocessed and included in our atlases.
(**B**) t-SNE of the meta head atlas containing more than 1 million cells/nuclei with cell types defined by unique colors.
(**C**) t-SNEs showing the distribution of cells expressing epithelial (*grh*), muscle (*Mhc*), fat (*AkhR*) and glia (*repo*) marker genes (top), and t-SNEs of the proportional contribution of different source tissue types to each cluster – central brain, optic lobe, and whole head (bottom).
(**D**) t-SNE of the meta neuron atlas containing more than 700K cells/nuclei, generated by removing all non-neuronal cells/nuclei from the meta head atlas.
(**E**) t-SNEs showing distribution of cells expressing known marker genes for photoreceptors (*ninaA*), first-order interneurons downstream of photoreceptors (*ort*), chemosensory neurons (*Orco*) and mechanosensory neurons (*nompC*) (top), and t-SNEs of the proportional contribution of different source tissue types to each cluster – central brain, optic lobe, and whole head (bottom).
(**F**) t-SNE of meta neuron atlas showing transferred annotation labels from Janssens et al., 2022[S12] to the other datasets (top), and t-SNE of meta neuron atlas showing transferred annotation labels from Ozel et al., 2021[S9] to the other datasets (bottom). Cells without annotations are plotted in white.
(**G**) UMAP (left) and t-SNE (right) of the meta central brain neuron atlas containing 329,466 cells/nuclei, generated by removing all non-central brain cells/nuclei from the meta neuron atlas with unsupervised clustered cell types defined by unique colors.

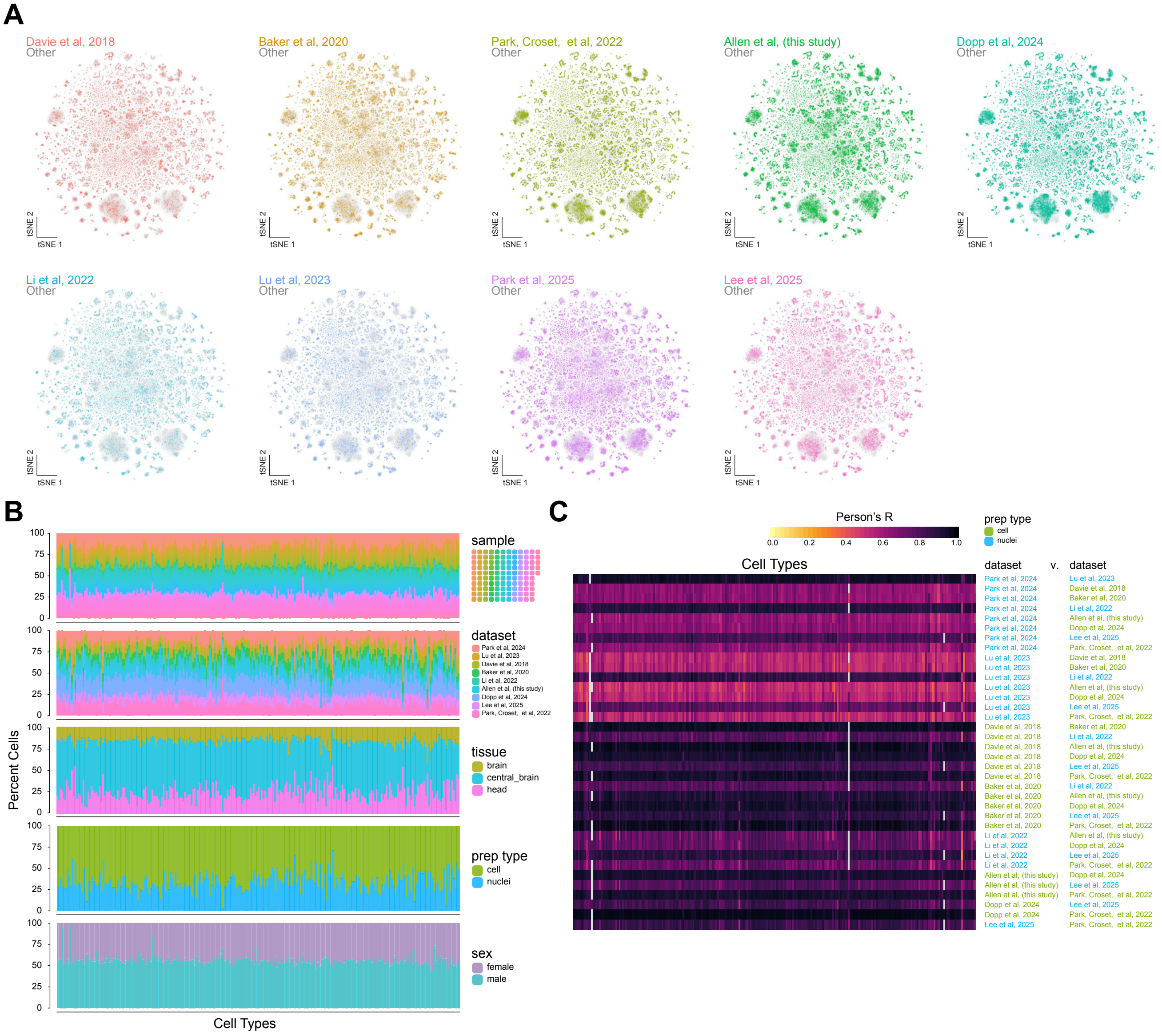

A
Davie et al, 2018
Other
Baker et al, 2020
Park, Croset, et al, 2022
Allen et al, (this study)
Dopp et al, 2024
Li et al, 2022
Lu et al, 2023
Park et al, 2025
Lee et al, 2025
tSNE 1
tSNE 2
B
Percent Cells
sample
dataset
Park et al, 2024
Lu et al, 2023
Davie et al, 2018
Baker et al, 2020
Li et al, 2022
Allen et al, (this study)
Dopp et al, 2024
Lee et al, 2025
Park, Croset, et al, 2022
tissue
brain
central_brain
head
prep type
cell
nuclei
sex
female
male
Cell Types
C
Person's R
0.0 0.2 0.4 0.6 0.8 1.0
Cell Types
dataset v. dataset

Figure S3

Figure S3. Integration of datasets in the central brain neuronal atlas.

Related to Figure 1.

(**A**) Dataset composition across studies contributing to the central brain meta-atlas. t-SNE plots showing the distribution of neurons from each contributing dataset within the integrated atlas. Cells from each study are overlaid in color; remaining neurons are shown in grey ("Other").
(**B**) Stacked bar plots showing the proportional contribution of samples, datasets, tissue, cell preparation, and sex across cell types.
(**C**) Heatmap showing Pearson's R correlation of dataset versus dataset across all cell types.

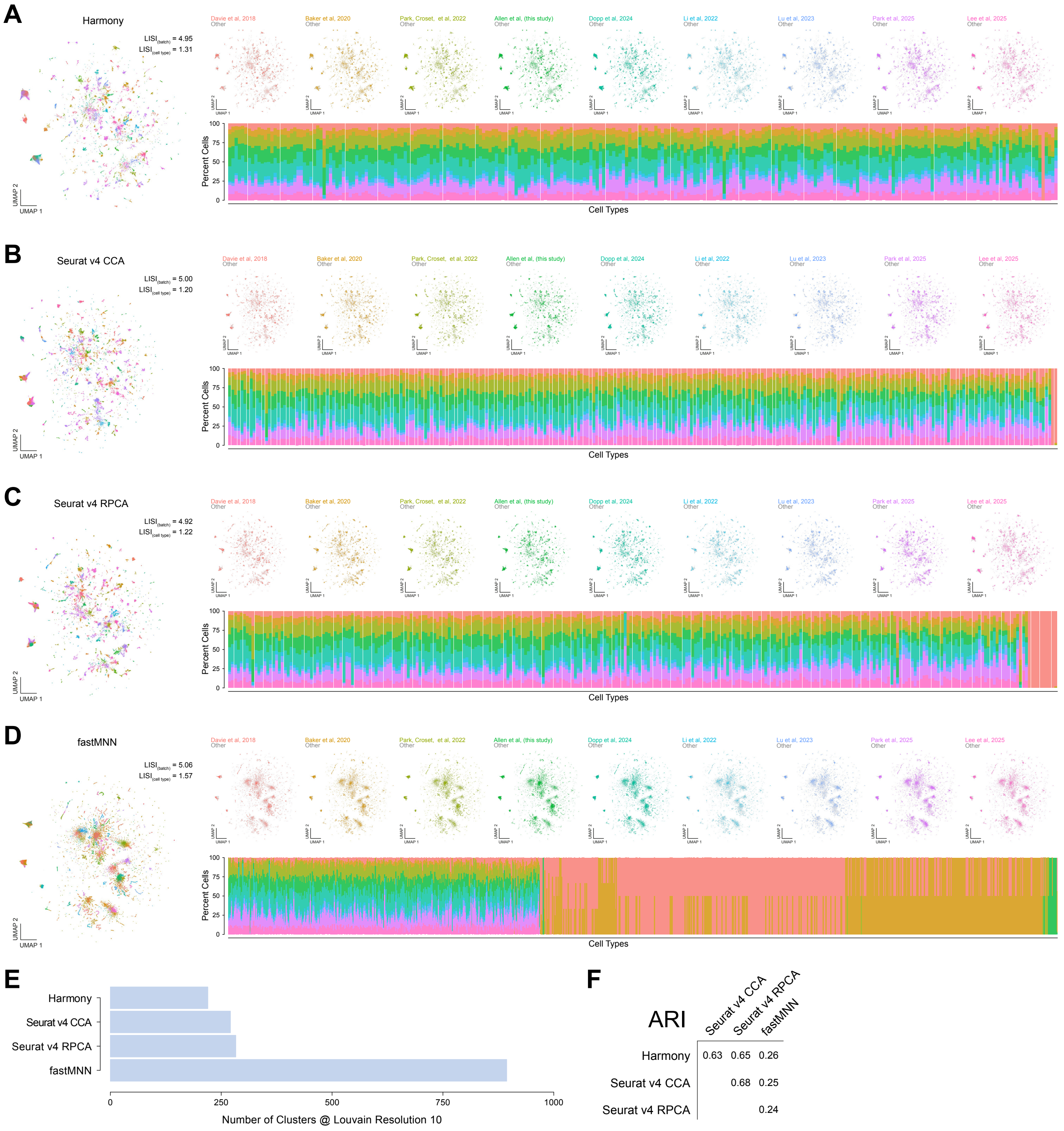
A
Harmony
LISI(batch) = 4.95
LISI(cell type) = 1.31
B
Seurat v4 CCA
LISI(batch) = 5.00
LISI(cell type) = 1.20
C
Seurat v4 RPCA
LISI(batch) = 4.92
LISI(cell type) = 1.22
D
fastMNN
LISI(batch) = 5.06
LISI(cell type) = 1.57
Davie et al, 2018
Baker et al, 2020
Park, Croset, et al, 2022
Allen et al, (this study)
Dopp et al, 2024
Li et al, 2022
Lu et al, 2023
Park et al, 2025
Lee et al, 2025
Other
UMAP 1
UMAP 2
Percent Cells
Cell Types
E
Harmony
Seurat v4 CCA
Seurat v4 RPCA
fastMNN
0
250
500
750
1000
Number of Clusters @ Louvain Resolution 10
F
ARI
Seurat v4 CCA
Seurat v4 RPCA
fastMNN
Harmony 0.63 0.65 0.26
Seurat v4 CCA 0.68 0.25
Seurat v4 RPCA 0.24

Figure S4

Figure S4. Comparison of data integration methods for the central brain neuronal atlas.

Related to Figure 1.

(**A**-**D**) Evaluation of four integration strategies: Harmony (**A**), Seurat v4 CCA (**B**), Seurat v4 RPCA (**C**), and fastMNN (**D**). For each method, UMAPs of integrated datasets are shown (left), dataset-specific contributions (middle), and dataset composition across unsupervised clustered cell types (stacked bar plots, bottom). Integration quality is assessed by dataset mixing and preservation of cell type structure; mean LISI (Local Inverse Simpson's Index) scores for batch mixing (batch) and cell type separation (cell type) are reported.
(**E**) Number of clusters detected at Louvain resolution 10 for each integration method.
(**F**) Adjusted Rand Index (ARI) between cell type clusters generated by each method, quantifying clustering consistency between methods.

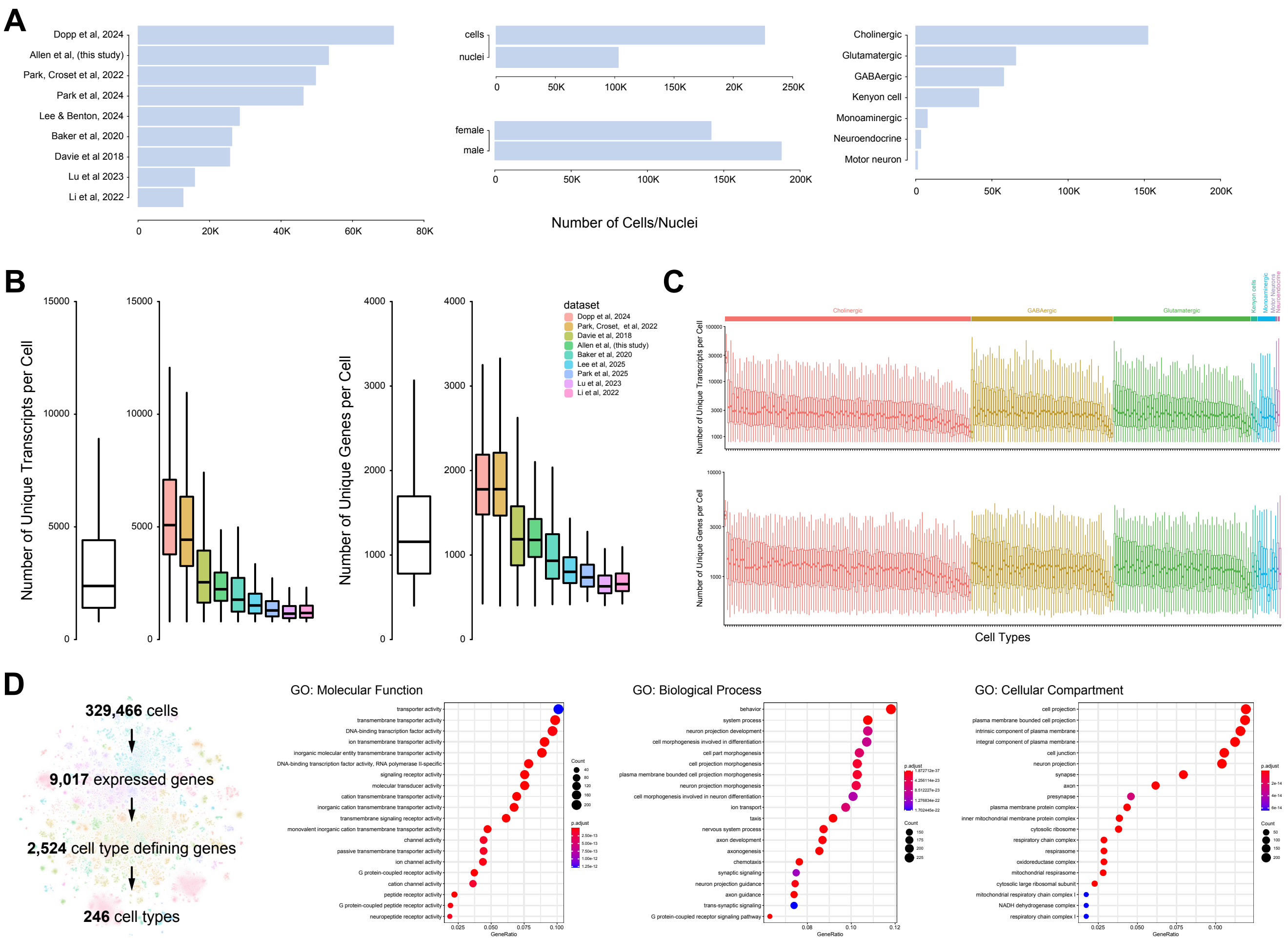
A
Dopp et al, 2024
Allen et al, (this study)
Park, Croset et al, 2022
Park et al, 2024
Lee & Benton, 2024
Baker et al, 2020
Davie et al 2018
Lu et al 2023
Li et al, 2022
cells
nuclei
female
male
Cholinergic
Glutamatergic
GABAergic
Kenyon cell
Monoaminergic
Neuroendocrine
Motor neuron
Number of Cells/Nuclei
B
Number of Unique Transcripts per Cell
Number of Unique Genes per Cell
dataset
Dopp et al, 2024
Park, Croset, et al, 2022
Davie et al, 2018
Allen et al, (this study)
Baker et al, 2020
Lee et al, 2025
Park et al, 2025
Lu et al, 2023
Li et al, 2022
C
Cell Types
D
329,466 cells
9,017 expressed genes
2,524 cell type defining genes
246 cell types
GO: Molecular Function
GO: Biological Process
GO: Cellular Compartment
GeneRatio

Figure S5

Figure S5. Dataset composition and transcriptional complexity across the central brain neuron atlas.

Related to Figure 1.

(**A**) Bar plots showing the number of cells/nuclei represented for each dataset (left), between cells and nuclei and assigned female or male (middle) and within broad cell type classifications in the meta neuron atlas (right).
(**B**) Boxplot showing the number of unique transcripts per cell (left) across integrated and individual datasets and the number of unique genes detected per cell (right) across integrated and individual datasets.
(**C**) Boxplots displaying the number of unique transcripts (top) and unique genes (bottom) per cell across neuronal cell types.
(**D**) On the left, flow diagram of the central brain neuron atlas, showing the number of single cells profiled, expressed genes (total UMI ≥ 400, max UMI ≥ 4, see STAR Methods), cell type-defining genes (Bonferroni corrected Wilcoxon Signed-Rank Test, $\log_2(FC) > 0.05$, p_{adj}-value < 0.05), and the number of identified cell types. On the right, gene ontology (GO) enrichment analysis of cell type defining genes for molecular function, biological processes, and cellular compartments.

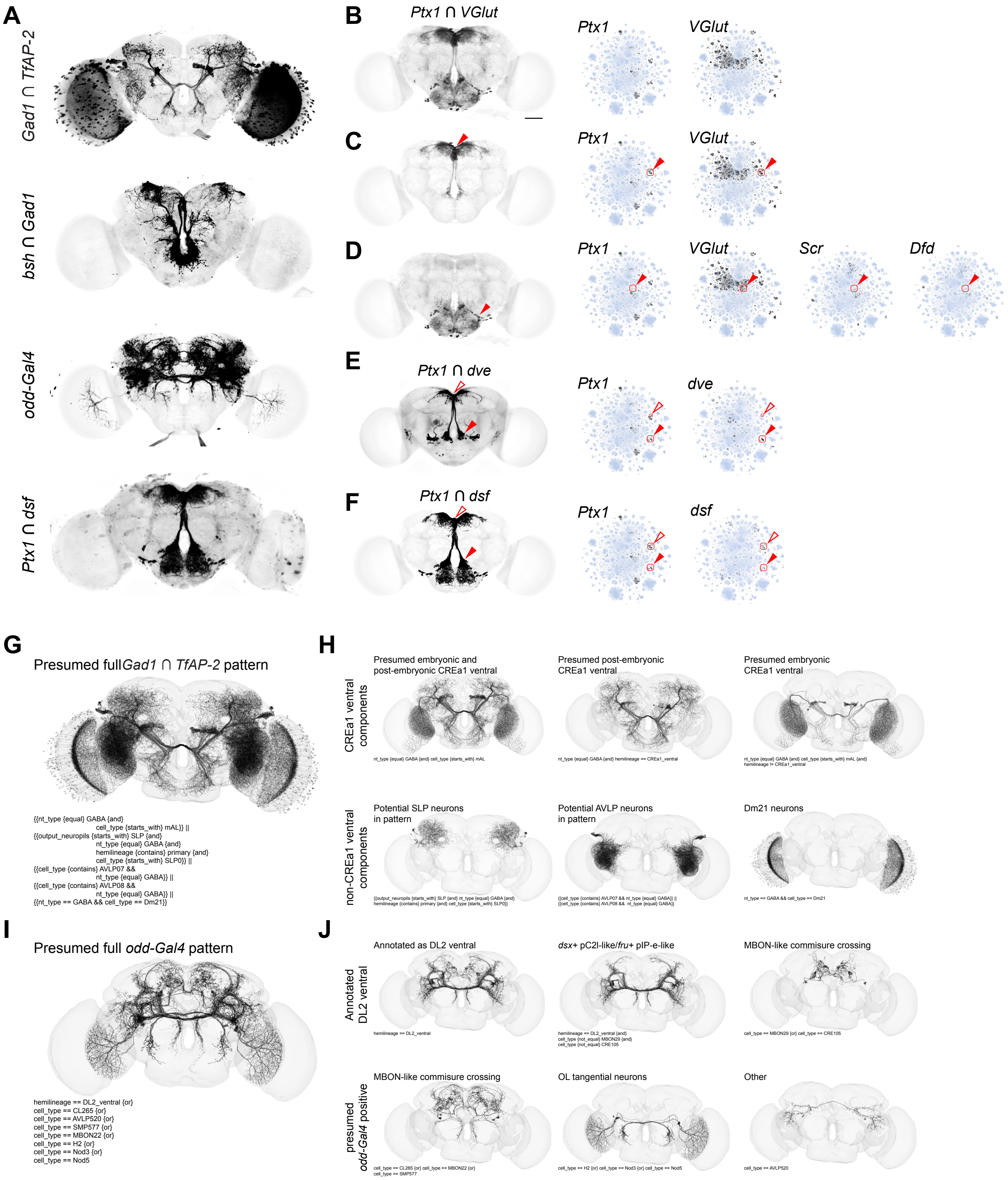

A
Gad1 ∩ TfAP-2
bsh ∩ Gad1
odd-Gal4
Ptx1 ∩ dsf
B
Ptx1 ∩ VGlut
Ptx1
VGlut
C
Ptx1
VGlut
D
Ptx1
VGlut
Scr
Dfd
E
Ptx1 ∩ dve
Ptx1
dve
F
Ptx1 ∩ dsf
Ptx1
dsf
G
Presumed full Gad1 ∩ TfAP-2 pattern
{{nt_type {equal} GABA {and}
cell_type {starts_with} mAL}} ||
{{output_neuropils {starts_with} SLP {and}
nt_type {equal} GABA {and}
hemilineage {contains} primary {and}
cell_type {starts_with} SLP0}} ||
{{cell_type {contains} AVLP07 &&
nt_type {equal} GABA}} ||
{{cell_type {contains} AVLP08 &&
nt_type {equal} GABA}} ||
{{nt_type == GABA && cell_type == Dm21}}
H
CREa1 ventral components
Presumed embryonic and post-embryonic CREa1 ventral
nt_type {equal} GABA {and} cell_type {starts_with} mAL
Presumed post-embryonic CREa1 ventral
nt_type {equal} GABA {and} hemilineage == CREa1_ventral
Presumed embryonic CREa1 ventral
nt_type {equal} GABA {and} cell_type {starts_with} mAL {and}
hemilineage != CREa1_ventral
non-CREa1 ventral components
Potential SLP neurons in pattern
{{output_neuropils {starts_with} SLP {and} nt_type {equal} GABA {and}
hemilineage {contains} primary {and} cell_type {starts_with} SLP0}}
Potential AVLP neurons in pattern
{{cell_type {contains} AVLP07 && nt_type {equal} GABA}} ||
{{cell_type {contains} AVLP08 && nt_type {equal} GABA}}
Dm21 neurons
nt_type == GABA && cell_type == Dm21
I
Presumed full odd-Gal4 pattern
hemilineage == DL2_ventral {or}
cell_type == CL265 {or}
cell_type == AVLP520 {or}
cell_type == SMP577 {or}
cell_type == MBON22 {or}
cell_type == H2 {or}
cell_type == Nod3 {or}
cell_type == Nod5
J
Annotated DL2 ventral
Annotated as DL2 ventral
hemilineage == DL2_ventral
dsx+ pC2l-like/fru+ pIP-e-like
hemilineage == DL2_ventral {and}
cell_type {not_equal} MBON29 {and}
cell_type {not_equal} CRE105
MBON-like commisure crossing
cell_type == MBON29 {or} cell_type == CRE105
presumed odd-Gal4 positive
MBON-like commisure crossing
cell_type == CL265 {or} cell_type == MBON22 {or}
cell_type == SMP577
OL tangential neurons
cell_type == H2 {or} cell_type == Nod3 {or} cell_type == Nod5
Other
cell_type == AVLP520

Figure S6

Figure S6. Genetic intersections to identify SMPad1, CREa1 ventral, DM2 central, and DL2 ventral hemilineages.

Related to Figure 2.

(**A**) Unsegmented whole-mount immunofluorescence of central brains as seen in Figure 2B.
(**B**) Whole-mount immunofluorescence of a central brain showing expression of $VGlut^{DBD}$ intersected against $Ptx1^{AD}$ (left). t-SNEs (right) showing *Ptx1* and *VGlut* expression in the central brain neuron atlas.
(**C**) Cerebrum segmented *Ptx1* and *VGlut* intersection expression from panel B (left) and t-SNE plots (right) showing gene *Ptx1* and *VGlut* expression in the central brain neuron atlas. SMPad1 hemilineage cells are highlighted with a red box and a solid arrowhead.
(**D**) Gnathal ganglia segmented *Ptx1* and *VGlut* intersection expression from panel B (left) and t-SNEs (right) showing gene *Ptx1*, *VGlut*, *Scr*, and *Dfd* expression in the central brain neuron atlas. Gnathal ganglia located cells are highlighted with a red box and a solid arrowhead.
(**E**) Whole-mount immunofluorescence of a central brain showing expression of dve^{DBD} intersected against $Ptx1^{AD}$ (left). t-SNEs (right) showing *Ptx1* and *dve* expression in the central brain neuron atlas. Main cell type intersected is highlighted with a red box and a solid arrowhead, and a subset of SMPad1 hemilineage intersected highlighted with a red box and an empty red arrow.
(**F**) Whole-mount immunofluorescence of a central brain showing expression of dsf^{DBD} intersected against $Ptx1^{AD}$ and segmented for clarity (left). t-SNEs (right) showing *Ptx1* and *dsf* expression in the central brain neuron atlas. Main intersected cell types, FLAa3 (red boxes and solid arrowhead) and SMPad1 (red boxes and open arrowhead) hemilineages are highlighted.
(**G**) EM rendering of predicted *Gad1* and *TfAP-2* intersected neurons in the central brain with FlyWire identifiers (below).
(**H**) EM rendering of predicted CREa1 ventral hemilineage (top). Presumed post-embryonic and embryonic born neurons are shown separately. EM rendering of predicted non-CREa1 ventral components of *Gad1* and *TfAP-2* intersected neurons shown (bottom), including SLP, AVLP, and Dm21 neurons. FlyWire identifiers are shown for all cell types.
(**I**) EM rendering of predicted *odd-Gal4* expressing neurons in the central brain with FlyWire identifier (bottom).
(**J**) All predicted DL2 ventral neurons with *dsx*+ pC2l-like/*fru*+ pIP-e-like and MBON-like subsets of the hemilineage are shown (top). Additional predicted DL2 ventral neurons based on *odd-Gal4* expression, including MBON-like, optic lobe tangential, and other unannotated (bottom). FlyWire identifiers are shown for all cell types.

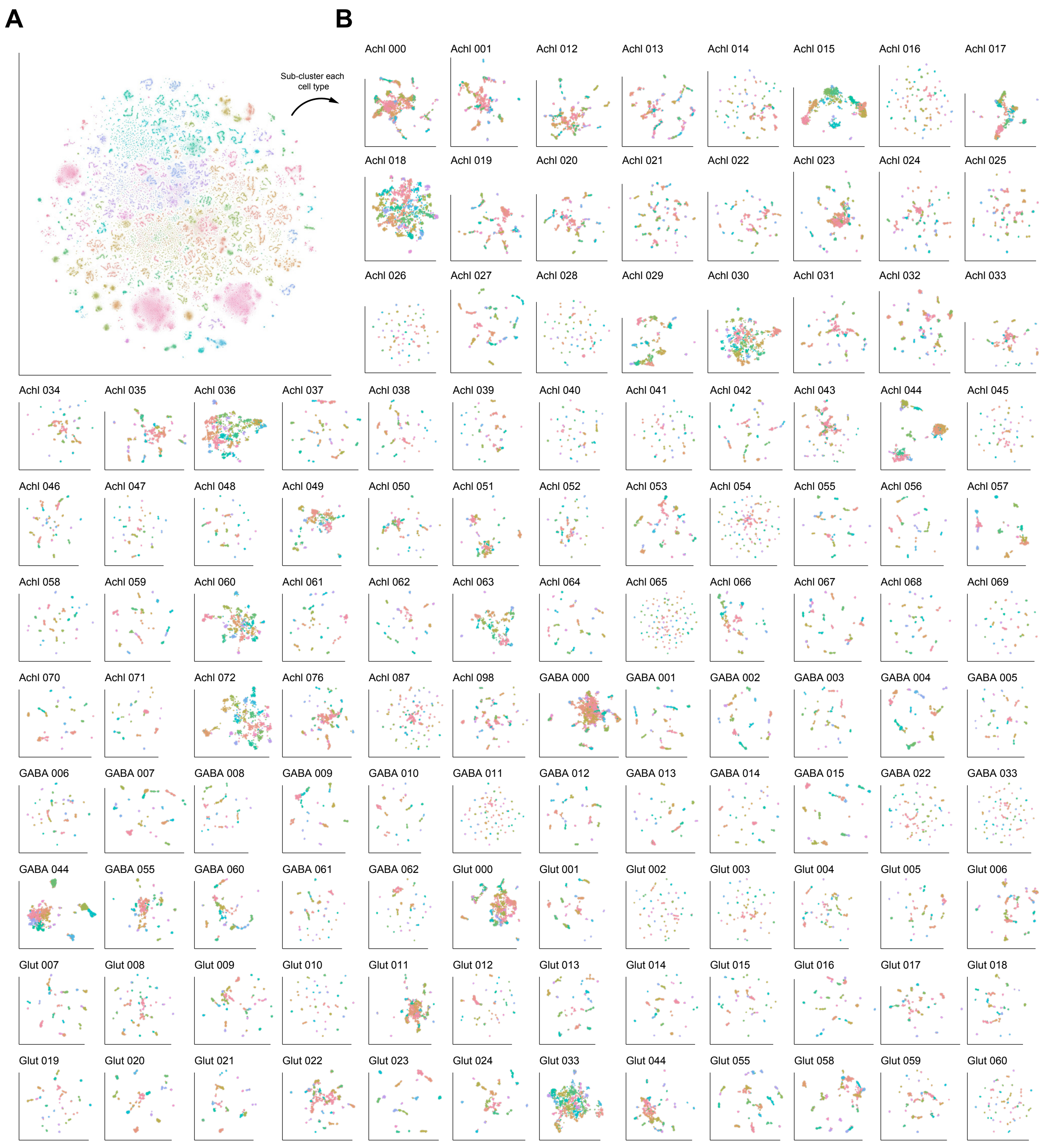
A
B
Sub-cluster each cell type
Achl 000
Achl 001
Achl 012
Achl 013
Achl 014
Achl 015
Achl 016
Achl 017
Achl 018
Achl 019
Achl 020
Achl 021
Achl 022
Achl 023
Achl 024
Achl 025
Achl 026
Achl 027
Achl 028
Achl 029
Achl 030
Achl 031
Achl 032
Achl 033
Achl 034
Achl 035
Achl 036
Achl 037
Achl 038
Achl 039
Achl 040
Achl 041
Achl 042
Achl 043
Achl 044
Achl 045
Achl 046
Achl 047
Achl 048
Achl 049
Achl 050
Achl 051
Achl 052
Achl 053
Achl 054
Achl 055
Achl 056
Achl 057
Achl 058
Achl 059
Achl 060
Achl 061
Achl 062
Achl 063
Achl 064
Achl 065
Achl 066
Achl 067
Achl 068
Achl 069
Achl 070
Achl 071
Achl 072
Achl 076
Achl 087
Achl 098
GABA 000
GABA 001
GABA 002
GABA 003
GABA 004
GABA 005
GABA 006
GABA 007
GABA 008
GABA 009
GABA 010
GABA 011
GABA 012
GABA 013
GABA 014
GABA 015
GABA 022
GABA 033
GABA 044
GABA 055
GABA 060
GABA 061
GABA 062
Glut 000
Glut 001
Glut 002
Glut 003
Glut 004
Glut 005
Glut 006
Glut 007
Glut 008
Glut 009
Glut 010
Glut 011
Glut 012
Glut 013
Glut 014
Glut 015
Glut 016
Glut 017
Glut 018
Glut 019
Glut 020
Glut 021
Glut 022
Glut 023
Glut 024
Glut 033
Glut 044
Glut 055
Glut 058
Glut 059
Glut 060

Figure S7

Figure S7. Subclustering of all central brain neuronal cell types.

Related to Figure 2.

(A) t-SNE of 329,466 central brain neurons representing 9.8x cellular depth coverage, colored by cell type.
(B) Each of the cell types in A was extracted, re-integrated, and re-clustered. UMAPs of the top 120 most numerous cell types (omitting Kenyon cells) are shown, highlighting the thousands of transcriptionally distinct neuronal subtypes that exist in the central brain. The first 20 PCs were used for the UMAP analyses, and Louvain clusters at resolution 2 are shown.

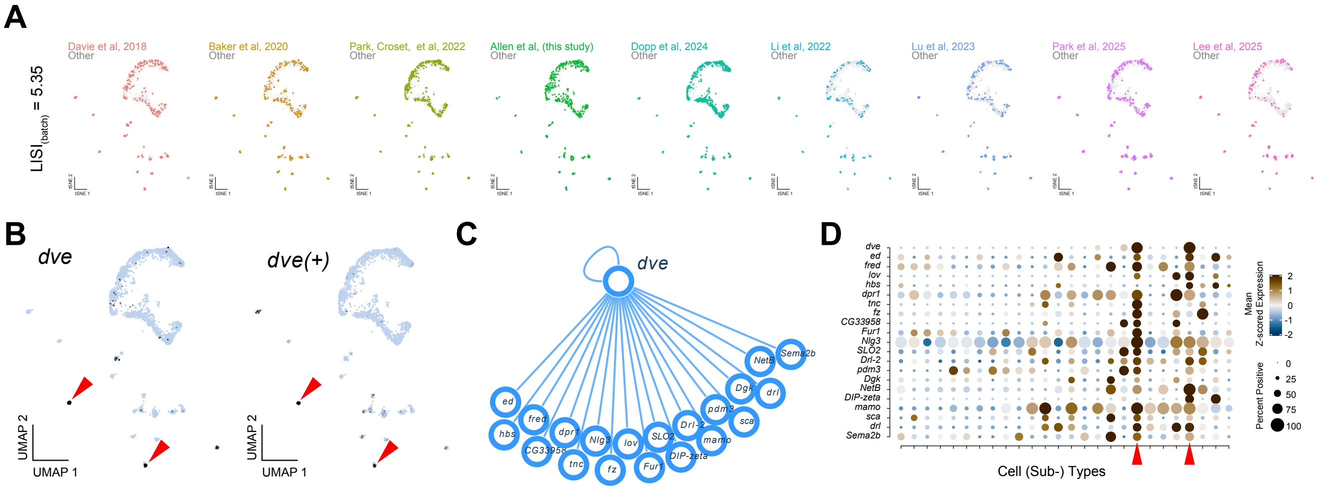
A
Davie et al, 2018
Other
Baker et al, 2020
Other
Park, Croset, et al, 2022
Other
Allen et al, (this study)
Other
Dopp et al, 2024
Other
Li et al, 2022
Other
Lu et al, 2023
Other
Park et al, 2025
Other
Lee et al, 2025
Other
LISI(batch) = 5.35
B
dve
dve(+)
UMAP 2
UMAP 1
C
dve
ed
fred
hbs
CG33958
dpr1
tnc
Nlg3
fz
lov
SLO2
Fur1
Drl-2
DIP-zeta
pdm3
mamo
Dgk
sca
NetB
drl
Sema2b
D
dve
ed
fred
lov
hbs
dpr1
tnc
fz
CG33958
Fur1
Nlg3
SLO2
Drl-2
pdm3
Dgk
NetB
DIP-zeta
mamo
sca
drl
Sema2b
Cell (Sub-) Types
Mean Z-scored Expression
2
1
0
-1
-2
Percent Positive
0
25
50
75
100

Figure S8

Figure S8. Gene regulatory network analysis reveals *dve* as a subtype-specific regulator within the SMPad1 hemilineage.

Related to Figure 2.

(**A**) UMAPs showing the distribution of SMPad1 neurons across contributing datasets. LISI score indicates the degree of batch mixing across datasets.
(**B**) UMAP showing *dve* gene expression (left) and *dve(+)* regulon (right) within the SMPad1 hemilineage; red arrowheads highlight subtype-specific *dve*-expressing populations.
(**C**) Predicted regulatory targets of Dve within the SMPad1 hemilineage based on SCENIC analysis.
(**D**) Dot plot of *dve* and its predicted targets across SMPad1 hemilineage subtypes; red arrowheads highlight subtype-specific *dve*-expressing populations.

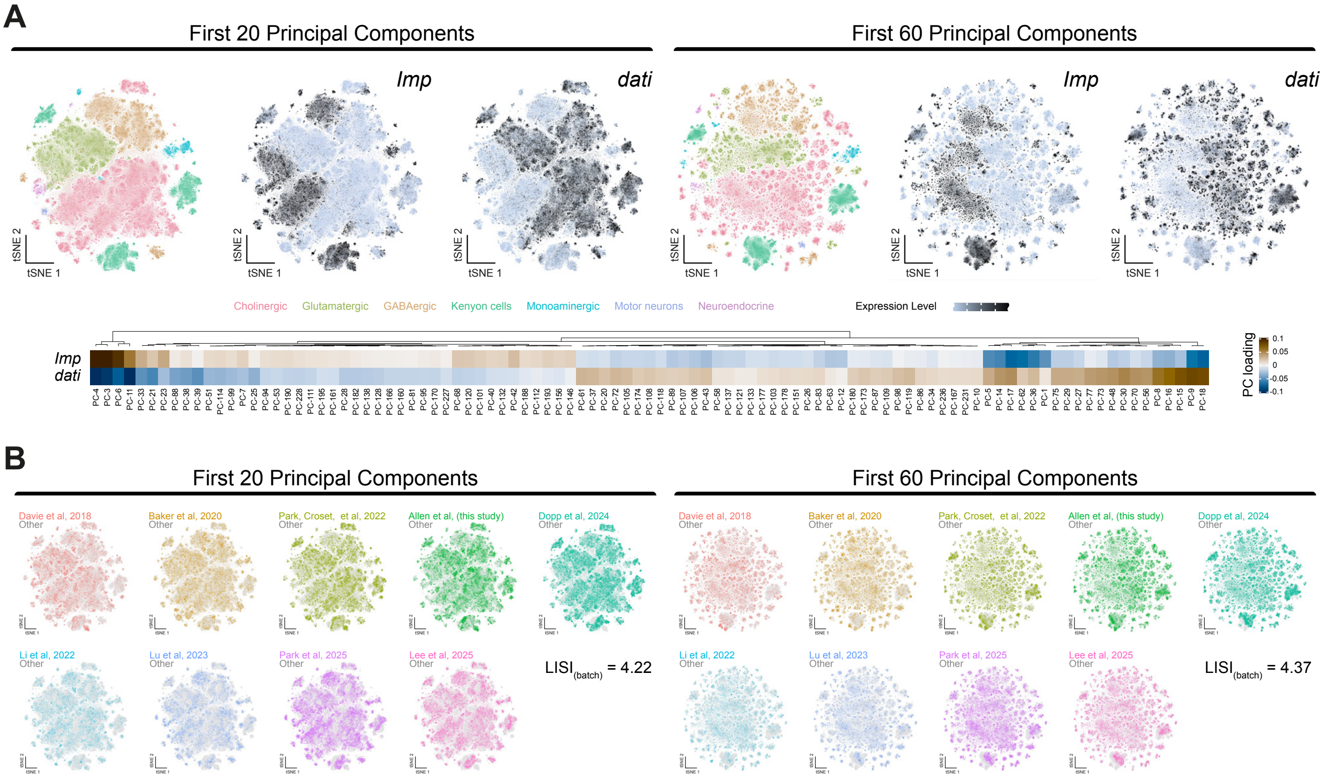
A
First 20 Principal Components
First 60 Principal Components
Imp
dati
tSNE 1
tSNE 2
Cholinergic
Glutamatergic
GABAergic
Kenyon cells
Monoaminergic
Motor neurons
Neuroendocrine
Expression Level
PC loading
0.1
0.05
0
-0.05
-0.1
B
First 20 Principal Components
First 60 Principal Components
Davie et al, 2018
Baker et al, 2020
Park, Croset, et al, 2022
Allen et al, (this study)
Dopp et al, 2024
Li et al, 2022
Lu et al, 2023
Park et al, 2025
Lee et al, 2025
Other
LISI(batch) = 4.22
LISI(batch) = 4.37

Figure S9

Figure S9. Early- versus late-born neurons exhibit distinct transcriptional profiles.

Related to Figure 3.

(**A**) Effects of a varied number of principal components on t-SNE morphology of central brain neurons. (Left) t-SNEs using the first 20 principal components, labeled by broad cell types or *Imp* and *dati* expression. (Right) t-SNEs using the first 60 principal components, labeled by broad cell types or *Imp* and *dati* expression. Separation of the early-born and late-born neurons is constant, and punctate versus serpentine t-SNE morphologies emerge with increased principal components. Heatmap of top *Imp* and *dati* loadings on principal components (bottom), where at least one had an absolute value greater than 0.01 (bottom), demonstrating *Imp* and *dati'*s contribution to many principal components.
(**B**) Batch-corrected t-SNE plots of early- and late-born neurons using 20 (left) or 60 (right) principal components, colored by contributing dataset. LISI score indicates the degree of batch mixing across datasets.

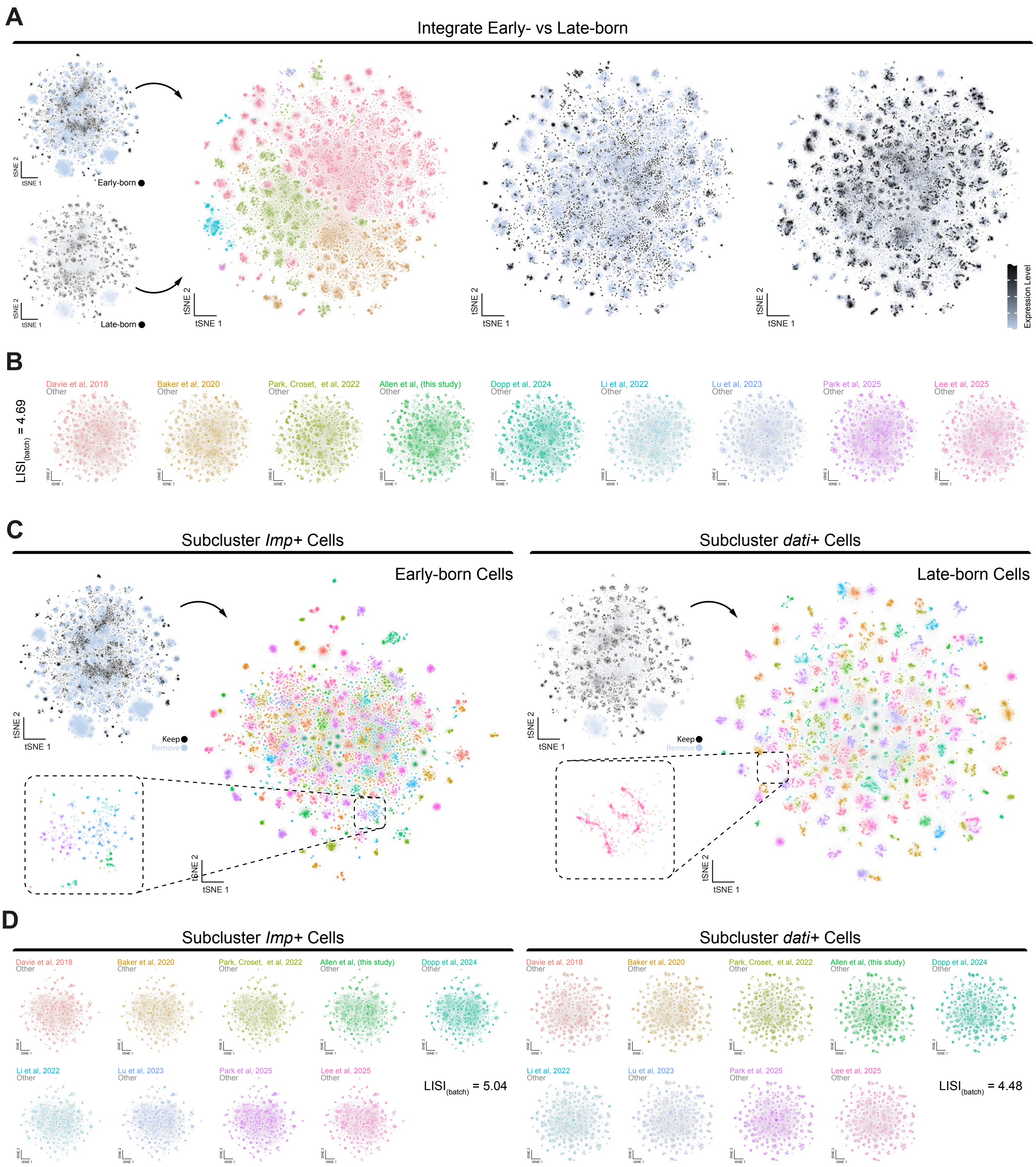
A
Integrate Early- vs Late-born
Early-born
Late-born
tSNE 1
tSNE 2
Expression Level
B
$LISI_{(batch)}$ = 4.69
Davie et al, 2018
Baker et al, 2020
Park, Croset, et al, 2022
Allen et al, (this study)
Dopp et al, 2024
Li et al, 2022
Lu et al, 2023
Park et al, 2025
Lee et al, 2025
Other
C
Subcluster Imp+ Cells
Subcluster dati+ Cells
Early-born Cells
Late-born Cells
Keep
Remove
D
Subcluster Imp+ Cells
Subcluster dati+ Cells
$LISI_{(batch)}$ = 5.04
$LISI_{(batch)}$ = 4.48

Figure S10

Figure S10. Subclustering of early- and late-born central brain neurons.

Related to Figure 3.

(**A**) Batch correction of early- and late-born neurons. t-SNEs of cells labeled as early-born and late-born before batch correction (left). Integrated clustering of all neuronal cell types following batch correction (middle). Expression of *Imp* and *dati* across the batch corrected atlas (right), showing the transcriptional separation of early- and late-born neurons remains.
(**B**) Integrated dataset colored by contributing study, showing consistent representation across the atlas. LISI score indicates the degree of batch mixing across datasets.
(**C**) Sub-clustering of early-born and late-born neuronal populations in the central brain. t-SNEs showing subclusterings of the early-born (*Imp*+) population (left) and late-born (*dati*+) population (right), with insets highlighting representative cluster morphologies. Punctate morphologies in the early-born sub-clustering and serpentine morphologies in the late-born sub-clustering remain.
(**D**) Dataset contributions for each subclustered atlas, confirming broad and balanced coverage across studies. LISI score indicates the degree of batch mixing across datasets.

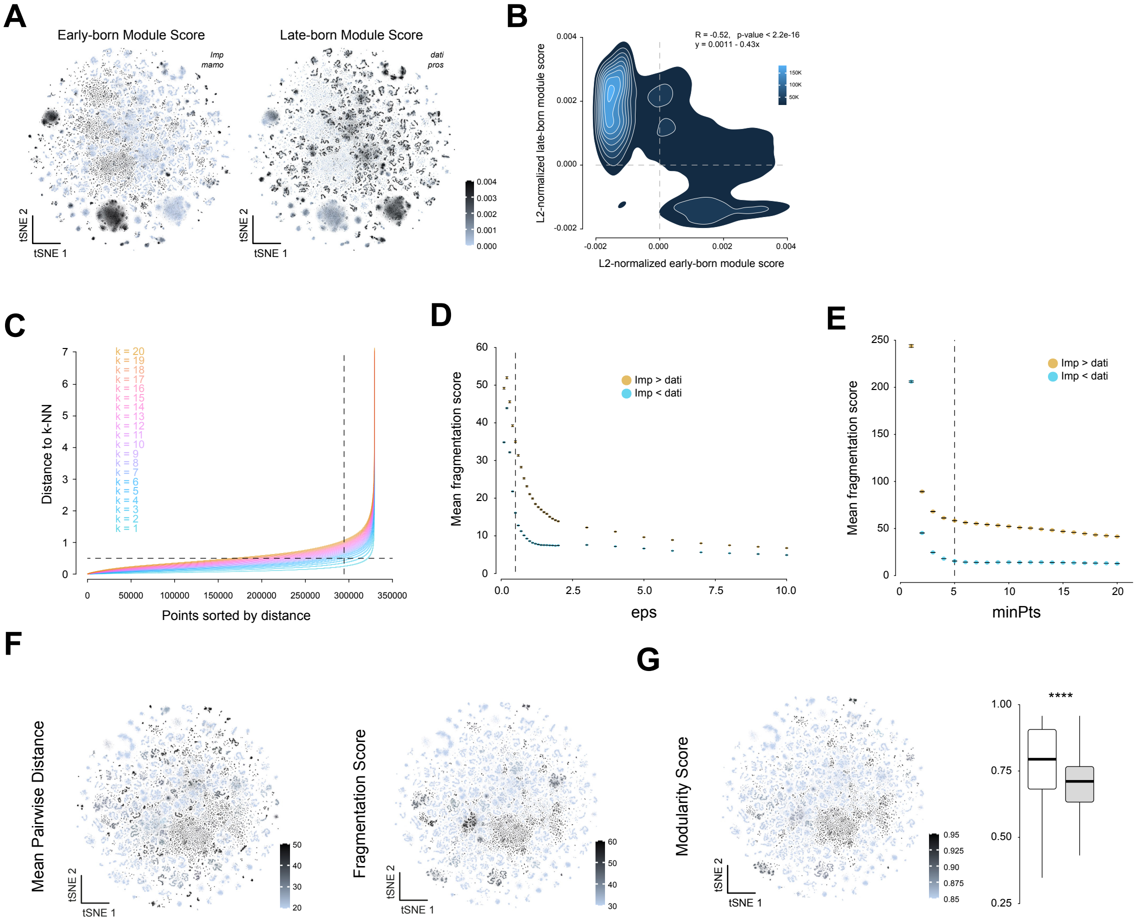
A
Early-born Module Score
Late-born Module Score
Imp
mamo
dati
pros
tSNE 2
tSNE 1
B
R = -0.52, p-value < 2.2e-16
y = 0.0011 - 0.43x
L2-normalized late-born module score
L2-normalized early-born module score
C
Distance to k-NN
Points sorted by distance
D
Mean fragmentation score
eps
Imp > dati
Imp < dati
E
minPts
F
Mean Pairwise Distance
Fragmentation Score
G
Modularity Score
****

Figure S11

Figure S11. Early- and late-born neurons exhibit distinct spatial organization in transcriptomic space.

Related to Figure 3.

(**A**) t-SNEs of early-born module score (*Imp*+, *mamo*+) and late-born module score (*dati*+, *pros*+) neurons in the adult central brain neuron atlas.
(**B**) A 2D density plot showing the distribution of central brain neurons based on their early-born and late-born module scores. A strong negative correlation is observed between the two scores, indicating that neurons with high expression of early-born module genes tend to show low expression of late-born module genes and vice versa.
(**C**) Distance (in t-SNE space) to k^{th}-nearest-neighbour (k-NN) for a range of k and sorted distance. Most cells have their 4th-NN within a distance of 0.5 (dashed lines).
(**D**) Relationship between the radius of the epsilon neighbourhood (eps) values against the mean fragmentation scores (±95% confidence interval) between early- and late-born cells, showing repeated differences in embedding structure between neuronal types of different developmental age, independent of dbscan parameters.
(**E**) Relationship between the radius of the number of neighbourhood members (minPts) values against the mean fragmentation scores (±95% confidence interval) between early- and late-born cells, showing repeated differences in embedding structure between neuronal types of different developmental age independent of dbscan parameters.
(**F**) Spatial properties of neuronal clusters. t-SNEs colored by mean pairwise distance (left) and fragmentation score (right), highlighting morphological differences in cluster organization.
(**G**) t-SNE visualisation of modularity scores across cell types (left) and box plot comparing modularity scores of early- and late-born cells (right), indicating greater modularity among early-born neurons (Bonferroni corrected Wilcoxon Signed-Rank Test, ****$p < 0.0001$).

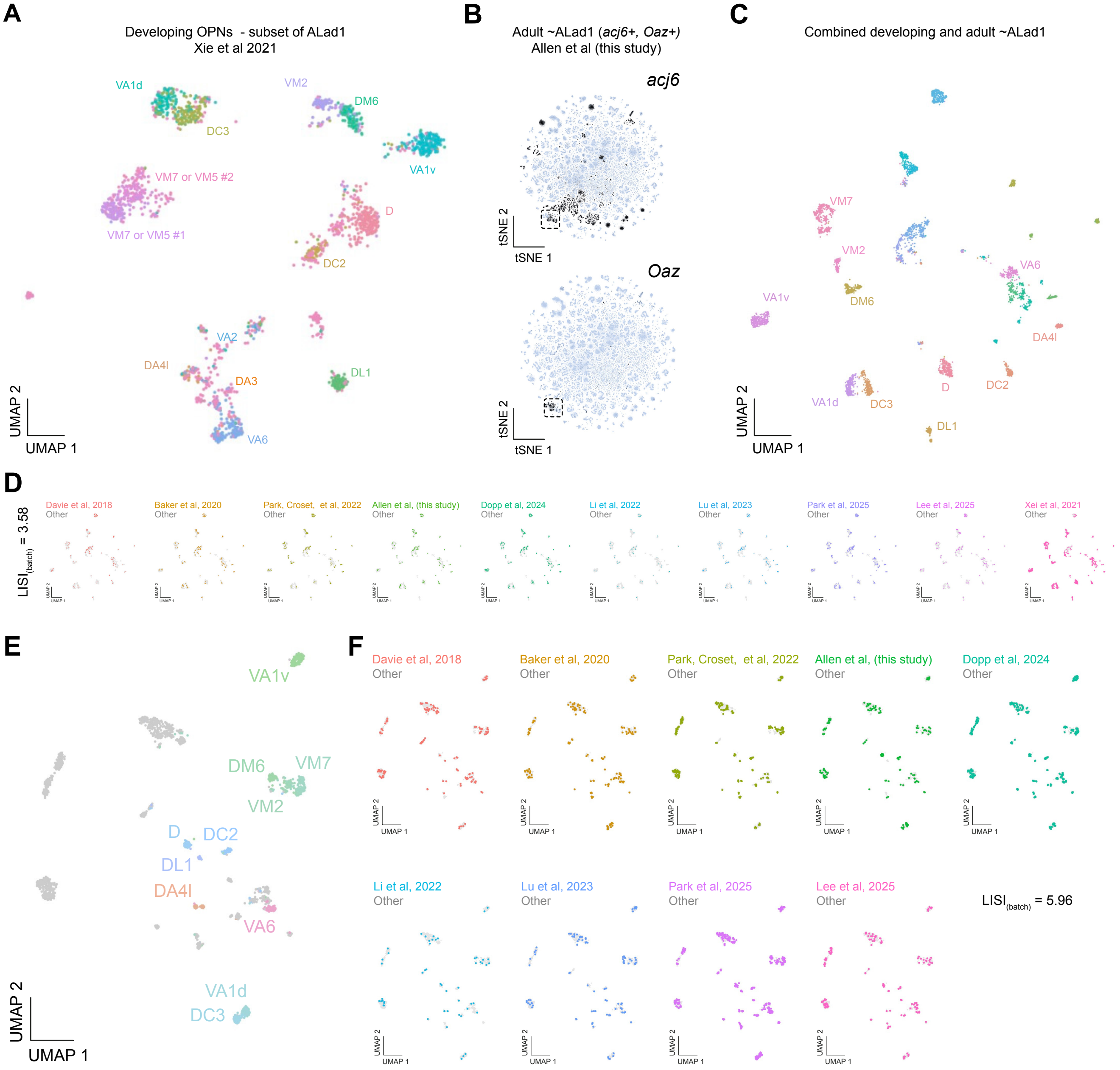

A
Developing OPNs - subset of ALad1
Xie et al 2021
VA1d
DC3
VM2
DM6
VA1v
VM7 or VM5 #2
VM7 or VM5 #1
D
DC2
VA2
DA4l
DA3
DL1
VA6
UMAP 2
UMAP 1
B
Adult ~ALad1 (acj6+, Oaz+)
Allen et al (this study)
acj6
Oaz
tSNE 2
tSNE 1
C
Combined developing and adult ~ALad1
VM7
VM2
DM6
VA1v
VA6
DA4l
D
DC2
VA1d
DC3
DL1
UMAP 2
UMAP 1
D
LISI(batch) = 3.58
Davie et al, 2018
Baker et al, 2020
Park, Croset, et al, 2022
Allen et al, (this study)
Dopp et al, 2024
Li et al, 2022
Lu et al, 2023
Park et al, 2025
Lee et al, 2025
Xei et al, 2021
Other
E
VA1v
DM6
VM7
VM2
D
DC2
DL1
DA4l
VA6
VA1d
DC3
UMAP 2
UMAP 1
F
Davie et al, 2018
Baker et al, 2020
Park, Croset, et al, 2022
Allen et al, (this study)
Dopp et al, 2024
Li et al, 2022
Lu et al, 2023
Park et al, 2025
Lee et al, 2025
Other
LISI(batch) = 5.96

Figure S12

Figure S12. ALad1 hemilineage identification and sub-clustering.

Related to Figure 4.

(**A**) UMAP plot of the developing OPN subset of ALad1. Reprocessed data originally from Xie et al., 2021[S13].
(**B**) t-SNE plots showing the ALad1 hemilineage marker genes *acj6* and *Oaz* expression in the central brain neuron atlas. The intersected cells type is highlighted with a dashed black box.
(**C**) UMAP plot of combined developing and adult ALad1 hemilineage.
(**D**) UMAP plots showing contribution of individual datasets in (C). LISI score indicates the degree of batch mixing across datasets.
(**E**) Annotated UMAP of adult ALad1 hemilineage, labeled by subtypes.
(**F**) UMAP plots showing contribution of individual datasets in (E). LISI score indicates the degree of batch mixing across datasets.

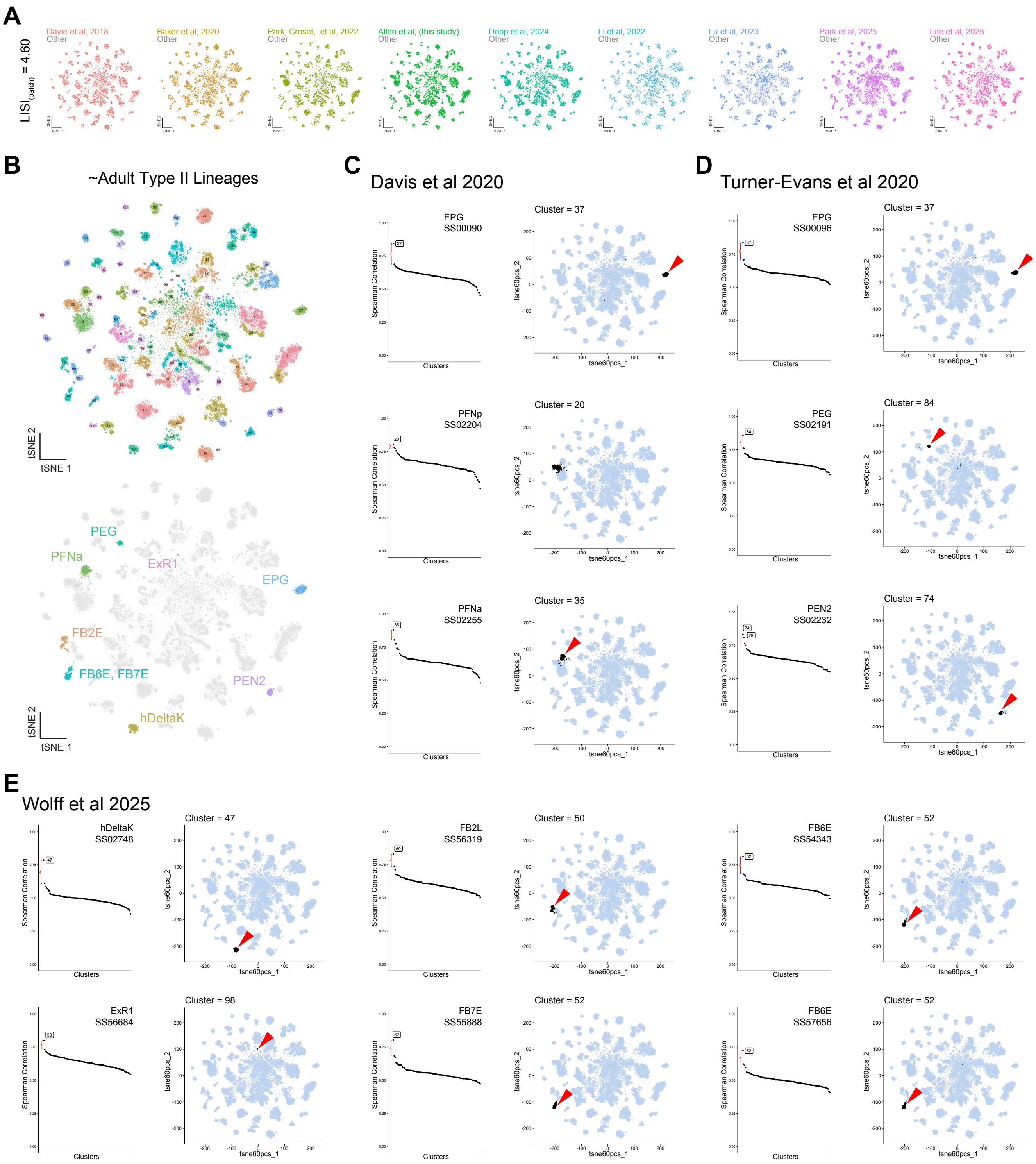
A
Davie et al, 2018
Baker et al, 2020
Park, Croset, et al, 2022
Allen et al, (this study)
Dopp et al, 2024
Li et al, 2022
Lu et al, 2023
Park et al, 2025
Lee et al, 2025
Other
LISI(batch) = 4.60
B
~Adult Type II Lineages
tSNE 1
tSNE 2
PEG
PFNa
ExR1
EPG
FB2E
FB6E, FB7E
PEN2
hDeltaK
C
Davis et al 2020
EPG SS00090
Cluster = 37
PFNp SS02204
Cluster = 20
PFNa SS02255
Cluster = 35
D
Turner-Evans et al 2020
EPG SS00096
Cluster = 37
PEG SS02191
Cluster = 84
PEN2 SS02232
Cluster = 74
E
Wolff et al 2025
hDeltaK SS02748
Cluster = 47
FB2L SS56319
Cluster = 50
FB6E SS54343
Cluster = 52
ExR1 SS56684
Cluster = 98
FB7E SS55888
Cluster = 52
FB6E SS57656
Cluster = 52
Spearman Correlation
Clusters
tsne60pcs_1
tsne60pcs_2

Figure S13

Figure S13. Cell type annotations across the type II neuroblast lineages atlas.

Related to Figure 5.

(**A**) Dataset composition across studies contributing to the type II neuroblast lineages atlas. t-SNE plots showing the distribution of neurons from each contributing dataset within the integrated atlas. Cells from each study are overlaid in color; remaining neurons are shown in grey ("Other"). LISI score indicates the degree of batch mixing across datasets.
(**B**) t-SNE of type II neuroblast lineage atlas with cell types defined by unique colors and numbers (top) and with cell types annotated based on bulk-seq correlations (bottom).
(**C**-**E**). Spearman correlation analysis of cell types in the adult type II atlas compared to available bulk sequencing data, showing the cluster number with the highest correlation (left) and the corresponding cluster in the type II adult atlas (right). Correlations using stable split (SS) labeled, FAC sorted, bulk sequencing data from (**C**) Davis et al., 2020[S14], (**D**) Turner-Evans et al., 2020[S15] and (**E**) Wolff et al., 2025[S16].

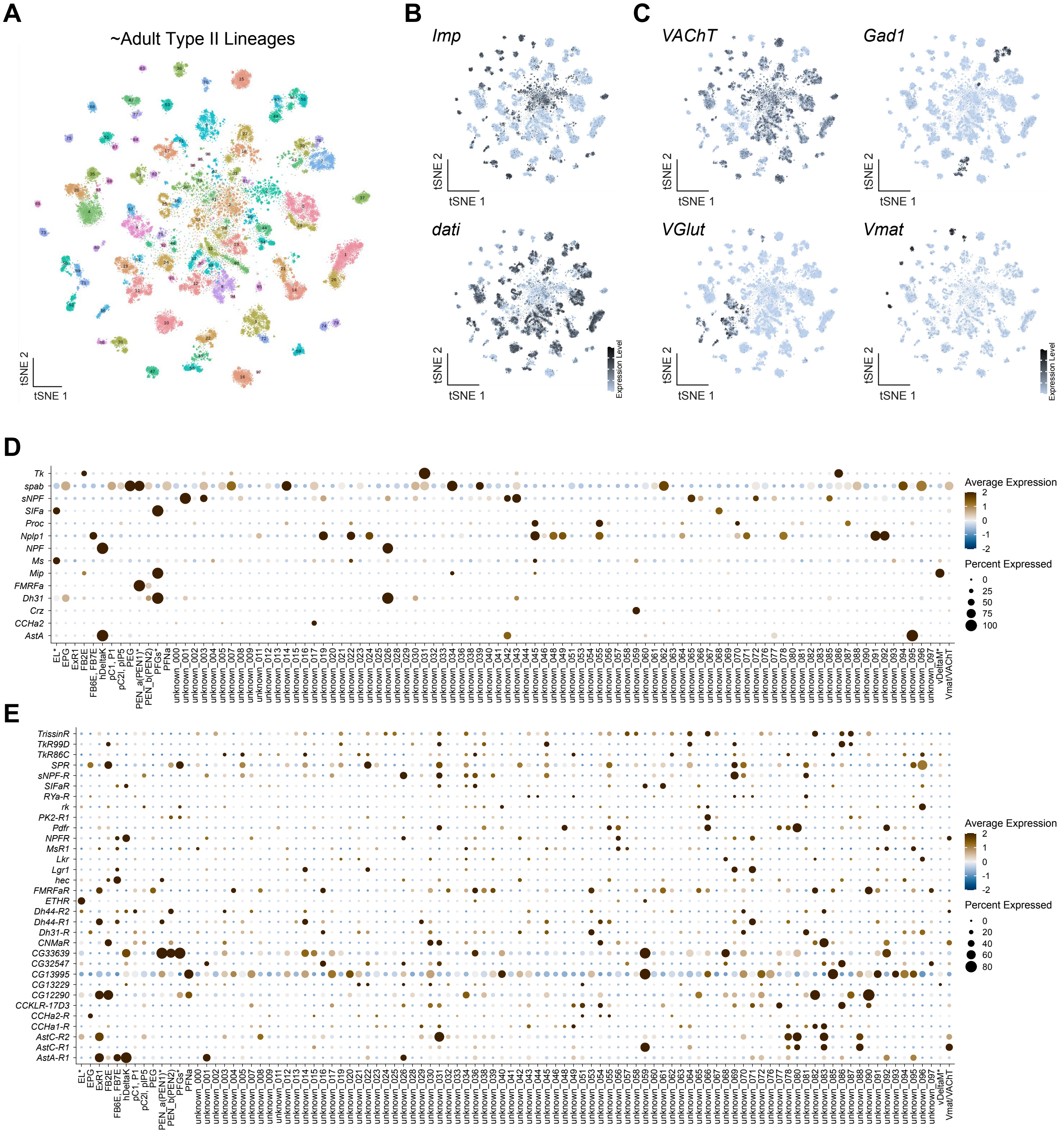
A
~Adult Type II Lineages
tSNE 2
tSNE 1
B
Imp
dati
C
VAChT
Gad1
VGlut
Vmat
Expression Level
D
Tk
spab
sNPF
SIFa
Proc
Nplp1
NPF
Ms
Mip
FMRFa
Dh31
Crz
CCHa2
AstA
Average Expression
Percent Expressed
E
TrissinR
TkR99D
TkR86C
SPR
sNPF-R
SIFaR
RYa-R
rk
PK2-R1
Pdfr
NPFR
MsR1
Lkr
Lgr1
hec
FMRFaR
ETHR
Dh44-R2
Dh44-R1
Dh31-R
CNMaR
CG33639
CG32547
CG13995
CG13229
CG12290
CCKLR-17D3
CCHa2-R
CCHa1-R
AstC-R2
AstC-R1
AstA-R1
Average Expression
Percent Expressed

Figure S14

Figure S14. Broad annotations and neuropeptide and neuropeptide receptor expression across the type II neuroblast lineages atlas.

Related to Figure 5.

(**A**) t-SNE of re-clustered type II neuroblast lineage atlas with cell types defined by unique colors and numbers.
(**B**) t-SNEs showing the expression of *Imp* (top) and *dati* (bottom) across the type II atlas.
(**C**) t-SNEs showing the expression of neurotransmitter marker genes across the type II atlas.
(**D**) Dot plot of neuropeptide expression across all type II-derived cell types.
(**E**) Dot plot of neuropeptide receptor expression across all type II-derived cell types.

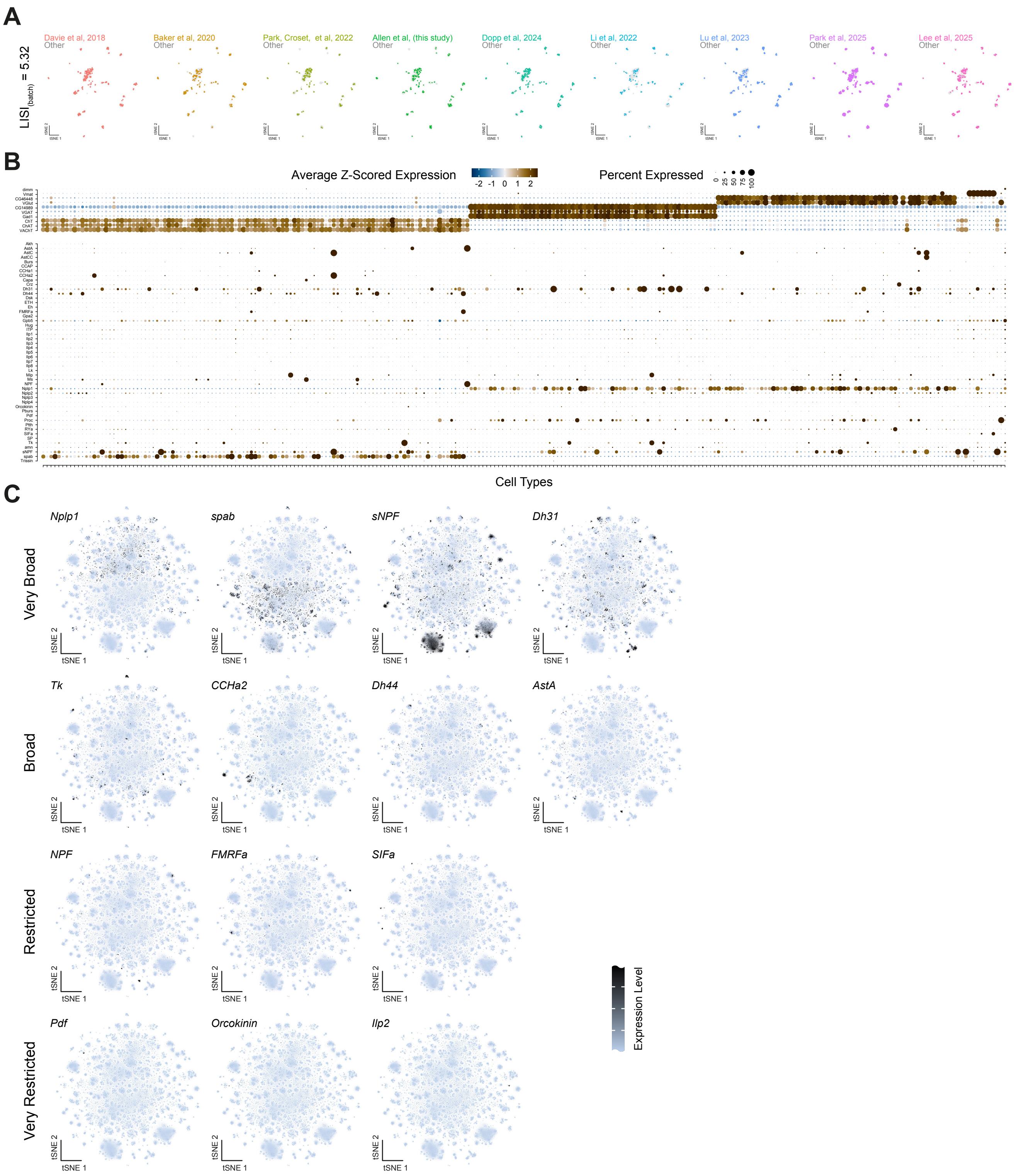
A
Davie et al, 2018
Baker et al, 2020
Park, Croset, et al, 2022
Allen et al, (this study)
Dopp et al, 2024
Li et al, 2022
Lu et al, 2023
Park et al, 2025
Lee et al, 2025
Other
LISI(batch) = 5.32
tSNE 1
tSNE 2
B
Average Z-Scored Expression
-2 -1 0 1 2
Percent Expressed
0 25 50 75 100
Cell Types
C
Very Broad
Nplp1
spab
sNPF
Dh31
Broad
Tk
CCHa2
Dh44
AstA
Restricted
NPF
FMRFa
SIFa
Very Restricted
Pdf
Orcokinin
Ilp2
Expression Level

Figure S15

Figure S15. Neuropeptide expression across the central brain.

Related to Figure 6.

(**A**) Dataset composition across studies contributing to neuroendocrine atlas. t-SNE plots showing the distribution of neurons from each contributing dataset within the neuroendocrine atlas. Cells from each study are overlaid in color; remaining neurons are shown in grey ("Other").
(**B**) Dot plot of broad cell type identifying genes (above) and all neuropeptide genes (below) across all cell types in the central brain. *Nplp1* is expressed broadly across glutamatergic and GABAergic cell types, while *spab* is expressed broadly across cholinergic cell types.
(**C**) t-SNEs showing examples of categories of neuropeptide gene expression across the central brain: very broadly expressed (*Nplp1*, *spab*, *sNPF*, *Dh31*), broad (*Tk*, *CCHa2*, *Dh44*, *AstA*), restricted (*NPF*, *FMRFa*, *SIFa*) and very restricted (*Pdf*, *Orckokinin*, *Ilp2*).

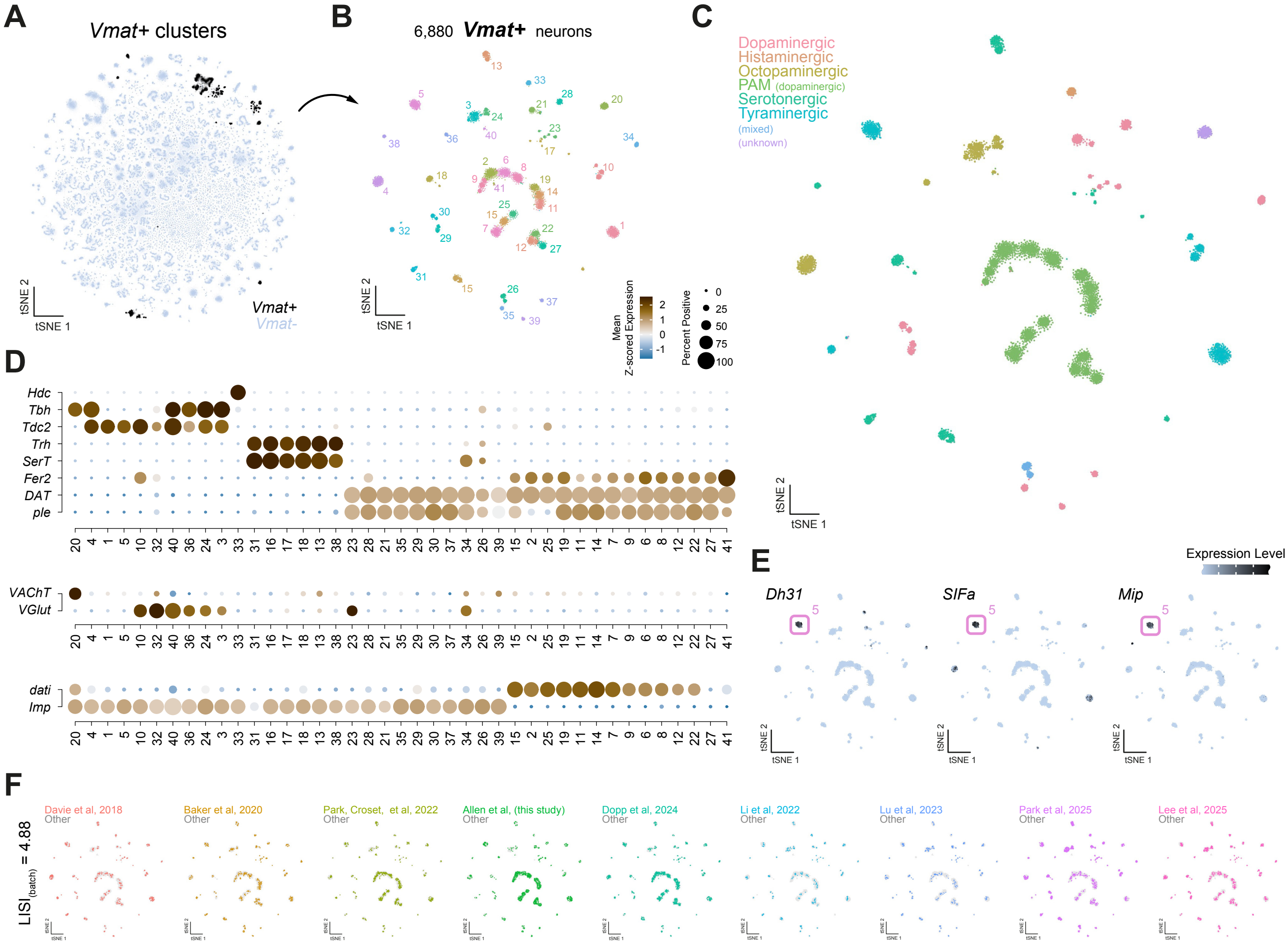
A
Vmat+ clusters
Vmat+
Vmat-
B
6,880 Vmat+ neurons
C
Dopaminergic
Histaminergic
Octopaminergic
PAM (dopaminergic)
Serotonergic
Tyraminergic
(mixed)
(unknown)
D
Mean Z-scored Expression
Percent Positive
Hdc
Tbh
Tdc2
Trh
SerT
Fer2
DAT
ple
VAChT
VGlut
dati
Imp
E
Expression Level
Dh31
SIFa
Mip
F
LISI(batch) = 4.88
Davie et al, 2018
Baker et al, 2020
Park, Croset, et al, 2022
Allen et al, (this study)
Dopp et al, 2024
Li et al, 2022
Lu et al, 2023
Park et al, 2025
Lee et al, 2025
Other

Figure S16

Figure S16. Transcriptional diversity of central brain monoaminergic cell types.

Related to Figure 6.

(**A**) t-SNE highlighting monoaminergic cell types based on the expression of the *Vesicular monoamine transporter* (*Vmat*) gene.
(**B**) t-SNE showing sub-clustering analysis of monoaminergic cell types. Transcriptionally distinct cell types are differentially colored and numbered.
(**C**) Central brain monoaminergic atlas annotated based on monoamine usage.
(**D**) Dot plot of biomarkers across monoaminergic cell types. Monoaminergic markers (top) *Hdc* labels histaminergic neurons, *Tbh* labels octopaminergic neurons, *Tdc-2* labels tyraminergic and octopaminergic neurons, *Trh* and *SerT* label serotonergic neurons, *DAT* and *ple* label dopaminergic neurons. The TF *Fer2* labels the protocerebral anterior medial (PAM) subset of dopamine neurons. Fast-acting neurotransmitter markers (middle) *VAChT* labels cholinergic and *VGlut* glutamatergic neurons. The birth order markers (below) *Imp* labels early-born and *dati* labels late-born neurons.
(**E**) t-SNEs showing example of neuropeptide co-expression of *Dh31*, *SIFa*, and *Mip* in tyraminergic subtype 5.
(**F**) Dataset composition across studies contributing to central brain monaminergic atlas. LISI score indicates the degree of batch mixing across datasets.

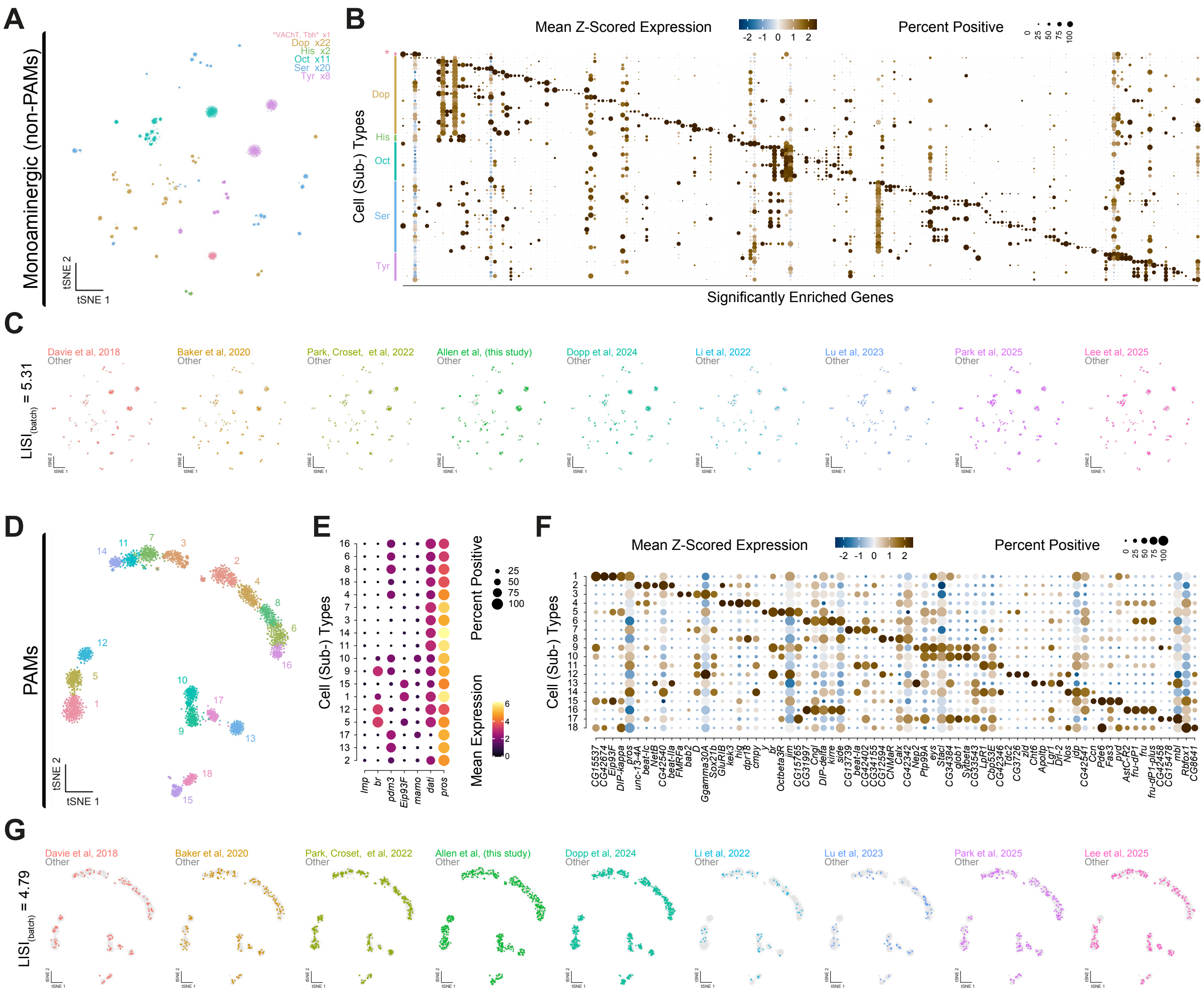
A
Monoaminergic (non-PAMs)
VAChT, Tbh x1
Dop x22
His x2
Oct x11
Ser x20
Tyr x8
B
Mean Z-Scored Expression
Percent Positive
Cell (Sub-) Types
Significantly Enriched Genes
C
LISI(batch) = 5.31
Davie et al, 2018
Baker et al, 2020
Park, Croset, et al, 2022
Allen et al, (this study)
Dopp et al, 2024
Li et al, 2022
Lu et al, 2023
Park et al, 2025
Lee et al, 2025
Other
D
PAMs
E
Cell (Sub-) Types
Percent Positive
Mean Expression
F
Mean Z-Scored Expression
Percent Positive
Cell (Sub-) Types
G
LISI(batch) = 4.79

Figure S17

Figure S17. Transcriptional diversity of central brain PAM and non-PAM cell types.

Related to Figure 6.

(**A**) t-SNE showing sub-clustering analysis of *Imp*+ monoaminergic cell types (i.e. PAMs removed). This analysis identified 64 transcriptionally distinct subtypes. Given the estimated 9.8x depth of coverage of cell types in this atlas, many of these clusters would correspond to subtypes with only 1-3 neurons per hemibrain.
(**B**) Dot plot of top genes defining monoaminergic, non-PAM, cell types.
(**C**) Dataset composition across studies contributing to monoaminergic, non-PAM, cell types. LISI score indicates the degree of batch mixing across datasets.
(**D**) t-SNE showing sub-clustering analysis of *Imp*- monoaminergic cell types (i.e. dopaminergic PAMs). Transcriptionally distinct cell types are differentially colored and numbered. This analysis identified 18 transcriptionally distinct subtypes.
(**E**) Dot plot of key developmental birth-order-defining genes across PAM subtypes.
(**F**) Dot plot of top genes defining PAM subtypes.
(**G**) Dataset composition across studies contributing to PAM subtypes. LISI score indicates the degree of batch mixing across datasets.

Supplementary References

S1. Davie, K., Janssens, J., Koldere, D., De Waegeneer, M., Pech, U., Kreft, L., Aibar, S., Makhzami, S., Christiaens, V., Bravo Gonzalez-Blas, C., et al. (2018). A Single-Cell Transcriptome Atlas of the Aging Drosophila Brain. Cell *174*, 982-998 e920. 10.1016/j.cell.2018.05.057.

S2. Baker, B.M., Mokashi, S.S., Shankar, V., Hatfield, J.S., Hannah, R.C., Mackay, T.F.C., and Anholt, R.R.H. (2021). The Drosophila brain on cocaine at single-cell resolution. Genome Res *31*, 1927-1937. 10.1101/gr.268037.120.

S3. Park, A., Croset, V., Otto, N., Agarwal, D., Treiber, C.D., Meschi, E., Sims, D., and Waddell, S. (2022). Gliotransmission of D-serine promotes thirst-directed behaviors in Drosophila. Curr Biol *32*, 3952-3970 e3958. 10.1016/j.cub.2022.07.038.

S4. Dopp, J., Ortega, A., Davie, K., Poovathingal, S., Baz, E.S., and Liu, S. (2024). Single-cell transcriptomics reveals that glial cells integrate homeostatic and circadian processes to drive sleep-wake cycles. Nat Neurosci *27*, 359-372. 10.1038/s41593-023-01549-4.

S5. Lee, D., Shahandeh, M.P., Abuin, L., and Benton, R. (2025). Comparative single-cell transcriptomic atlases of drosophilid brains suggest glial evolution during ecological adaptation. PLoS Biol *23*, e3003120. 10.1371/journal.pbio.3003120.

S6. Li, H., Janssens, J., De Waegeneer, M., Kolluru, S.S., Davie, K., Gardeux, V., Saelens, W., David, F.P.A., Brbic, M., Spanier, K., et al. (2022). Fly Cell Atlas: A single-nucleus transcriptomic atlas of the adult fruit fly. Science *375*, eabk2432. 10.1126/science.abk2432.

S7. Lu, T.C., Brbic, M., Park, Y.J., Jackson, T., Chen, J., Kolluru, S.S., Qi, Y., Katheder, N.S., Cai, X.T., Lee, S., et al. (2023). Aging Fly Cell Atlas identifies exhaustive aging features at cellular resolution. Science *380*, eadg0934. 10.1126/science.adg0934.

S8. Park, Y.-J., Lu, T.-C., Jackson, T., Goodman, L.D., Ran, L., Chen, J., Liang, C.-Y., Harrison, E., Ko, C., Chen, X., et al. (2025). Distinct systemic impacts of Aβ42 and Tau revealed by whole-organism snRNA-seq. Neuron *113*, 2065-2082.e2068. 10.1016/j.neuron.2025.04.017.

S9. Ozel, M.N., Simon, F., Jafari, S., Holguera, I., Chen, Y.C., Benhra, N., El-Danaf, R.N., Kapuralin, K., Malin, J.A., Konstantinides, N., and Desplan, C. (2021). Neuronal diversity and convergence in a visual system developmental atlas. Nature *589*, 88-95. 10.1038/s41586-020-2879-3.

S10. Kurmangaliyev, Y.Z., Yoo, J., Valdes-Aleman, J., Sanfilippo, P., and Zipursky, S.L. (2020). Transcriptional Programs of Circuit Assembly in the Drosophila Visual System. Neuron *108*, 1045-1057 e1046. 10.1016/j.neuron.2020.10.006.

S11. Yeung, K., Bollepogu Raja, K.K., Shim, Y.K., Li, Y., Chen, R., and Mardon, G. (2022). Single cell RNA sequencing of the adult Drosophila eye reveals distinct clusters and novel marker genes for all major cell types. Commun Biol *5*, 1370. 10.1038/s42003-022-04337-1.

S12. Janssens, J., Aibar, S., Taskiran, II, Ismail, J.N., Gomez, A.E., Aughey, G., Spanier, K.I., De Rop, F.V., Gonzalez-Blas, C.B., Dionne, M., et al. (2022). Decoding gene regulation in the fly brain. Nature *601*, 630-636. 10.1038/s41586-021-04262-z.

S13. Xie, Q., Brbic, M., Horns, F., Kolluru, S.S., Jones, R.C., Li, J., Reddy, A.R., Xie, A., Kohani, S., Li, Z., et al. (2021). Temporal evolution of single-cell transcriptomes of Drosophila olfactory projection neurons. Elife *10*. 10.7554/eLife.63450.

S14. Davis, F.P., Nern, A., Picard, S., Reiser, M.B., Rubin, G.M., Eddy, S.R., and Henry, G.L. (2020). A genetic, genomic, and computational resource for exploring neural circuit function. Elife *9*. 10.7554/eLife.50901.

S15. Turner-Evans, D.B., Jensen, K.T., Ali, S., Paterson, T., Sheridan, A., Ray, R.P., Wolff, T., Lauritzen, J.S., Rubin, G.M., Bock, D.D., and Jayaraman, V. (2020). The Neuroanatomical Ultrastructure and Function of a Biological Ring Attractor. Neuron *108*, 145-163 e110. 10.1016/j.neuron.2020.08.006.

S16. Wolff, T., Eddison, M., Chen, N., Nern, A., Sundaramurthi, P., Sitaraman, D., and Rubin, G.M. (2025). Cell type-specific driver lines targeting the Drosophila central complex and their use to investigate neuropeptide expression and sleep regulation. ELife *14*.
